# Inoculation for Enteric Fever

**Published:** 1902-01-18

**Authors:** 


### INOCULATION FOR ENTERIC FEVER.

Major C. Birt,[1] R.A.M.C., has compared the mortality from enteric fever, and also the severity of the disease among the inoculated and the uninoculated. He says that of those who suffered from enteric fever in Harrismith, South Africa, from September 1900 to September 1901, 947 were uninoculated. There were 135 deaths among them, or a fatality of 14·25 per cent. During the same period there were 263 cases among persons who had been inoculated with typhoid vaccine, for the most part 6 to 18 months previously. Of these cases 18 proved fatal, or a fatality of 6·8 per cent. An analysis of some of the charts of both classes showed that the average duration of the pyrexia was less, the average maximum temperature recorded was lower, and the percentage of cases in which relapses occurred was considerably less among the inoculated than among the uninoculated. It is to be remembered that even a complete attack of enteric fever by no means of a certainty produces immunity, so that the results above given are better even than one might have expected.

[1] Brit. Med. Journ. Jan. 11.

